# 

**Published:** 1905-06-15

**Authors:** 


### CONTENTS.

Event and Comment - - - - - - - - - - - 271
Do Modern Alloys Shrink, - - - - By M. L. Ward, D.D.S. 276
The Treatment of Some Phases of Alveolar Abscesses, By Geo. W. Cook, B.S., D.D.S. 285
Replantation, Transplantation and Implantation, By J. F. Austin, D.D.S. 290
President's Address, - - - - - - By A. L. Legro, D.D.S. 296
A New Porcelain Crown, - - - By C. H. Worboys, D.D.S. 298
"Prophylaxis" and "Pyorrhoea Alveolaris," By C. M. Wright, D.D.S. 300
Forty-nine Cases of Artificial Dentures Swallowed, By Arthur D. Black, B. S., M.D., D.D.S. 304
Pulp Extirpation, - - - - By Elgin Mawhinney, D.D.S. 308
Announcements - - - - - - - - - - - - 309
Obituary - - - - - - - - - - - - - - 313
Indents - - - - - - - - - - - - - 314

